# The Effects of Statins on Prostate Cancer Patients Receiving Androgen Deprivation Therapy or Definitive Therapy: A Systematic Review and Meta-Analysis

**DOI:** 10.3390/ph15020131

**Published:** 2022-01-22

**Authors:** Yu-Chen Hou, Yu-Hsuan Shao

**Affiliations:** 1 Graduate Institute of Biomedical Informatics, College of Medical Science and Technology, Taipei Medical University, Taipei 10675, Taiwan; d610109002@tmu.edu.tw; 2 Clinical Big Data Research Center, Taipei Medical University Hospital, Taipei 10675, Taiwan

**Keywords:** statin, androgen deprivation therapy, definitive therapy, prostate cancer, meta-analysis, mortality, cancer-specific mortality

## Abstract

Mortality associated with statin use has been reported in prostate cancer (PCa) patients treated with androgen deprivation therapy (ADT) or definitive therapy in several observational studies, although the results have varied. This study aimed to analyze the association of statin use with all-cause mortality and cancer-specific mortality among PCa patients receiving ADT or definitive therapy as their primary treatment and to examine the effect of statin initiation (pre-ADT) timing on outcomes. A systematic literature search of PubMed, the Cochrane library, and Embase was conducted from database inception to 4 October 2021. In total, 12 eligible studies from 976 references were included in the final analysis. The results showed that statin use was associated with a significant reduction in the risks of all-cause mortality (hazard ratio (HR) = 0.73, 95% confidence interval (CI) = 0.64–0.84, *p* < 0.0001) and cancer-specific mortality (HR = 0.61, 95% CI = 0.49–0.77, *p* < 0.0001) in PCa patients receiving ADT. However, statin use before ADT initiation did not significantly lower the risk of all-cause mortality (HR = 0.87, 95% CI = 0.66–1.16, *p* = 0.35) or cancer-specific mortality (HR = 0.84, 95% CI = 0.62–1.13, *p* = 0.25) in advanced PCa patients receiving ADT. In contrast, statin use was not associated with a significantly reduced risk of all-cause mortality (HR = 0.69, 95% CI = 0.39–1.21, *p* = 0.20), but it was associated with a reduced risk of cancer-specific mortality (HR = 0.82, 95% CI = 0.68–0.98, *p* = 0.03) in PCa patients receiving definitive therapy. This review indicated that statin use in combination with ADT was correlated with better all-cause and cancer-specific mortality in PCa patients. However, the beneficial effect might not come from statin use before ADT initiation. In addition, statin use in combination with definitive therapy was correlated with a reduced risk of cancer-specific mortality in PCa patients. In the future, randomized controlled trials are needed to validate the efficacy of statin use in combination with primary treatment for PCa among PCa patients.

## 1. Introduction

Statins have been widely used for decades to decrease serum cholesterol levels and the risk of cardiovascular disease [1]. Most recently, statins have received considerable attention due to their potential anticancer properties [2]. Several in vitro studies showed that statins prevent the progression of prostate cancer (PCa) via cholesterol-dependent and cholesterol-independent mechanisms [3]. In addition, PCa patients are usually older, and a high proportion of patients coexist with hyperlipidemia treated with statins [4]. The anticancer effect of statins in these patients is of interest. However, PCa is heterogeneous. Some patients are diagnosed as low risk with a favorable outcome, but others have a more aggressive form of cancer [5]. Although many observational studies have investigated the effects of statins on several outcomes regarding cancer progression, the association between statin use and their roles in reducing mortality in PCa patients is not entirely confirmed [6,7,8,9]. Furthermore, experts have been skeptical about these observational studies due to the prevalent user effect [10].

PCa is one of the most common cancers in men, with an estimated incidence of 1.4 million cases, and it accounts for 14.1% of cancer cases diagnosed in men as of 2020 [11]. Definitive therapy, including radical prostatectomy and radiation therapy, is the most common primary treatment option for localized PCa [12]. Additionally, androgen deprivation therapy (ADT) is widely accepted as the initial treatment for metastatic PCa [13].

A few meta-analysis studies investigated this topic, but the results were inconclusive. One meta-analysis demonstrated that in PCa patients, pre-diagnostic and post-diagnostic users of statins were associated with lower risks of all-cause mortality and PCa-specific mortality [14]. Notably, another meta-analysis showed that the effect of statins on PCa progression might depend on the primary treatment modality [15]. Moreover, a very recent meta-analysis provided evidence that statin use in combination with ADT was associated with reduced risks of all-cause and PCa-specific mortality in PCa patients [16]. Thus, evidence demonstrating the effects of statins in patients receiving different treatment modalities is important for clinical practice. We conducted this meta-analysis of all studies to date that evaluated the association between statin use and mortality in patients with PCa receiving either definitive therapy (radical prostatectomy or radiation therapy) or ADT as their primary treatment. We also assessed the effect of statins on mortality in prevalent statin users in a group of PCa patients receiving ADT as their primary treatment.

## 2. Methods

### 2.1. Search Strategy and Study Eligibility

We performed a systematic literature search of PubMed, the Cochrane library, and Embase from their inception to 4 October 2021 to identify all studies that investigated the association between statin use and the mortality of PCa patients receiving ADT or definitive therapy as their primary treatment. Additionally, guided by the Preferred Reporting Items for Systematic Reviews and Meta-Analyses (PRISMA) [17], we also screened reference lists of retrieved articles. For database searches, terms related to PCa and statins were combined with ADT and definitive therapy. Full details of the search strategy for each database are reported in Supplementary Table S1.

Prospective cohort studies, retrospective cohort studies, and randomized controlled trials (RCTs) that involved PCa patients receiving ADT or definitive therapy combined with statin use were included. We excluded studies reporting the efficacy of statins without a control group, studies without a mortality outcome, and studies that lacked extractable outcome data. The outcomes of interest for our review were all-cause mortality and cancer-specific mortality. Two authors (Hou and Shao) independently checked the inclusion and exclusion criteria for each of the identified studies and resolved discrepancies by discussion.

### 2.2. Data Extraction and Quality Assessment

Two authors (Hou and Shao) independently extracted the following data from each study using a predefined data extraction form: (1) study information, including author, publication year, study design, country of participants, sample size, and length of follow-up; (2) characteristics of participants, including PCa stage, type of primary treatment, the time of statin initiation, and the percentage of PCa patients receiving ADT among PCa patients taking definitive therapy as their primary treatment; and (3) outcomes, including all-cause mortality and cancer-specific mortality, with details on statistical parameters, such as adjusted hazard ratios (HRs; aHRs).

The Newcastle–Ottawa Scale (NOS) was utilized to examine the quality of the included observational studies [18]. The NOS consists of three domains, namely, patient selection, comparability, and outcome. A score with a range of 0–9 was allocated to each study. A higher score indicates higher quality, and studies with a score of ≥7 were considered to be high-quality studies [19]. Two reviewers (Shao and Hou) independently evaluated the quality of the studies. Disagreements regarding quality assessment scores for each study were resolved by consensus.

### 2.3. Statistical Analysis

HRs with a 95% confidence interval (CI) for mortality outcomes were calculated by pooling study-specific-adjusted multivariate HRs using the inverse variance method in Review Manager for Windows (vers. 5.4). We first pooled study-specific HRs for the effect of statins on mortality outcomes in PCa patients receiving ADT as their primary treatment. Next, to evaluate whether the statin initiation time affected the association between statin use and mortality, we pooled study-specific HRs for the effect of statins on mortality outcomes in ADT patients who used statins before ADT initiation (pre-ADT, prevalent users). Last, we pooled study-specific HRs for the effect of statins on mortality outcomes in PCa patients receiving definitive therapy as their primary treatment. For any study providing separate risk estimates by different definitive therapies, we considered the estimates as different studies [8].

Between-study heterogeneity was assessed using both Cochrane’s Q-test and the I^2^ statistic [20]. A value of *p* < 0.01 for the Q-test or I^2^ > 50% was considered significant. If heterogeneity reached statistical significance, a random effects model was adopted; otherwise, a fixed effects model was adopted [21].

A sensitivity analysis was performed to evaluate the robustness of the association between statin use and mortality outcomes in PCa patients receiving definitive therapy. For all-cause mortality, we pooled the lowest HRs in each study together. Meanwhile, the highest HRs in each study were also pooled together in the sensitivity analysis. However, for cancer-specific mortality, only three studies reported HRs. These HRs were derived from three populations with different statin initiation times, so we pooled the highest HRs from each study in the main analysis. In contrast, the lowest HRs in each study were pooled together in the sensitivity analysis.

For all comparisons, visual inspection of funnel plots was used to assess potential publication bias [22].

## 3. Results and Discussion

### 3.1. Literature Search

**Figure 1:**
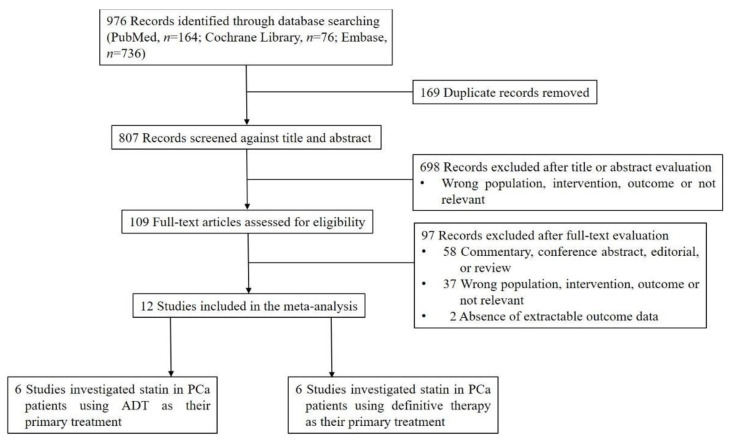
PRISMA flow diagram of the study selection process.

In total, 976 citations were identified through the electronic databases using the search strategy. Figure 1 summarizes the study selection process. Studies with the wrong population, intervention, or outcome were excluded. In addition, studies that were not relevant to assessing the effects of statin use among PCa patients or had no extractable outcome data, as well as commentaries, conference abstracts, editorials, and reviews, were excluded. Finally, 12 studies met the inclusion criteria for the current meta-analysis [8,9,23,24,25,26,27,28,29,30,31,32]. Six studies investigated the effects of statin use in PCa patients using ADT as their primary treatment [23,24,25,26,27,28]. The other six studies investigated the effects of statin use in PCa patients using definitive therapy as their primary treatment [8,9,29,30,31,32].

### 3.2. Characteristics of the Included Studies

The characteristics of the six studies that investigated the effects of statin use in PCa patients using ADT as their primary treatment are presented in Table 1. All six studies were retrospective cohort studies published between 2015 and 2021. They were conducted in Korea, Denmark, the United States, Taiwan, Canada, and Finland, with cohort sizes ranging between 171 and 87,346. The percentage of statin users ranged from 26.3% to 61%. In total, five studies included patients initiating statin use after a PCa diagnosis [24,25,26,27,28], and one study (by Jung et al.) included patients who initiated statin use before and after a PCa diagnosis [23]. Four studies reported both all-cause mortality and cancer-specific mortality [25,26,27,28], one only reported all-cause mortality [24], and one only reported cancer-specific mortality [23].

Furthermore, the characteristics of the six studies that investigated the effects of statin use in PCa patients receiving definitive therapy as their primary treatment are presented in Table 2. All six studies were retrospective cohort studies published between 2010 and 2021. Studies were conducted in the United States, Canada, Finland, and Taiwan, with cohort sizes ranging between 567 and 14,424. The percentage of statin users ranged from 23.73% to 46.79%. Primary treatment in the six studies included a radical prostatectomy, external beam radiotherapy, and brachytherapy. In addition to using definitive therapy as their primary treatment, patients in the studies may have received ADT as adjuvant or neoadjuvant therapy. Only one study (by Prabhu et al.) restricted patients to those without adjuvant hormone therapy [32]. All six studies had a median of at least 3.5 years of follow-up. One study reported both all-cause mortality and cancer-specific mortality [29], three reported only all-cause mortality [8,31,32], and two reported only cancer-specific mortality [9,30].

Due to no apparent asymmetry found by visual inspection of the funnel plots, no comparison in our meta-analysis demonstrated any significant publication bias.

**Table 1:** (a) Characteristics and (b) mortality outcomes of six studies investigating the effect of statin use in prostate cancer (PCa) patients receiving androgen deprivation therapy (ADT) as their primary treatment.

(a) Author, Year	Study Design, Years, Country	Study Characteristics	Treatment			Time of Statin Use	Follow-Up	Statin Users (*n*)	Non-Statin Users (*n*)	Mortality Outcomes
Jung et al. 2015 [23]	Retrospective cohort, 1997.1–2013.12, Korea	171 patients with metastatic PCa	ADT			Pre- and post-diagnostic	52 months (mean)	46	125	PCa-specific
Mikkelsen et al. 2017 [24]	Retrospective cohort, 2007.1.1–2013.12.31, Denmark	537 patients with advanced or metastatic PCa (65.9% metastatic PCa)	ADT, anti-androgens were used as flare protection only			Post-diagnostic and before ADT	5.7 years (median)	141	396	All-cause
Anderson-Carter et al. 2018 [25]	Retrospective cohort, 2000.1.1–2008.12.31, US	87,346 patients with advanced PCa	ADT for at least 6 months			Post-diagnostic	Until death or the end of the study (2016.5.31)	53,360	33,986	All-cause; PCa-specific
Wu et al. 2019 [26]	Retrospective cohort, 2008–2014, Taiwan	5749 patients with locally advanced and metastatic PCa (55% with metastatic PCa)	ADT			Post-diagnostic	3.6 years (mean)	2171	3578	All-cause; PCa-specific
Hamilton et al. 2021 [27]	Retrospective cohort, 1999.1–2005.11, Canada	1364 patients with advanced PCa with no distant metastasis	ADT			Post-diagnostic and before ADT	6.9 years (median)	585	779	All-cause; PCa-specific
Peltomaa et al. 2021 [28]	Retrospective cohort, 1996–2015, Finland	4428 patients with PCa (12.6% with metastatic PCa)	ADT			Post-diagnostic	6.3 years (median)	2544	1884	All-cause; PCa-specific
**(b) Author, Year**	**All-Cause Mortality**		**Cancer-Specific Mortality**			**Adjusted For**				
	**HR**	**95% CI**	***p***	**HR**	**95% CI**	***p***				
Jung et al. 2015 [23]	NR	NR	NR	0.41	0.25–0.66	<0.001	Age, hypertension, body-mass index, hypercholesterolemia, PSA level, and metastasis type (bone vs. bone and viscera)			
Mikkelsen et al. 2017 [24]	1.11	0.82–1.50	0.49	NR	NR	NR	Age, CCI score, Gleason score, clinical T-stage, PSA level, and metastatic status (M0 and M1)			
Anderson-Carter et al. 2018 [25]	0.66	0.63–0.68	<0.001	0.56	0.53–0.60	<0.001	Age, duration of ADT use, race, CCI score, Agent Orange exposure, year of the cancer diagnosis, PSA level, and Gleason score			
Wu et al. 2019 * [26]	0.75	0.68–0.82	NR	0.77	0.69–0.86	NR	Cancer stage, cancer grade, and year of the cancer diagnosis, and the use of metformin, NSAIDs, and aspirin			
Hamilton et al. 2021 [27]	0.64	0.53–0.78	<0.001	0.65	0.48–0.87	0.004	Age, time from RT to ADT, PSA level, and prior ADT use			
Peltomaa et al. 2021 ^†^ [28]	(1) 1.13 (2) 0.84	(1) 1.02–1.25 (2) 0.76–0.93	NR	(1) 1.12 (2) 0.82	(1) 0.96–1.31 (2) 0.69–0.96	NR	Age, tumor risk group (low, intermediate, high), randomization group (PSA screening or none), use of other medication (antidiabetic and antihypertensive drugs, NSAIDs), and whether participants received radiation therapy in addition to ADT			

Abbreviations: CCI, Charlson comorbidity index; CI, confidence interval; NR, not reported; NSAIDs, non-steroidal anti-inflammatory drugs; PSA, prostate-specific antigen; RT, radiotherapy. * In this study, the effect of statins on the risk of mortality was reported in both new statin users (*n* = 1562) and prevalent statin users (*n* = 609); statins initiated 1 year prior to the cancer diagnosis were prevalent users. For all-cause mortality and cancer-specific mortality, HRs are the same between the total population and prevalent users for both outcomes. † Associations between statin use and mortality outcomes were reported for different times of statin use, including (1) statin use before ADT initiation and (2) statin use after ADT initiation.

**Table 2:** (A) Characteristics and (B) mortality outcomes of six studies investigating the effect of statin use in prostate cancer (PCa) patients receiving definitive therapy as their primary treatment.

(A) Author, Year	Study Design, Years, Country	Study Characteristics		Primary Treatment	Time of Statin Use		Follow-Up	Statin users (*n*)	Non-statin Users (*n*)	ADT^†^	Mortality Outcomes
Katz et al. 2010 * [8]	Retrospective cohort, 1990–2003, USA	7042 patients with PCa (clinical stage including T1, T2, ≥T3)		RP or RT	Post-primary treatment		44 or 42 months (median)	1824	5218	(1) 14% (2) 15%	All-cause
Caon et al. 2014 [9]	Retrospective cohort, 2000.1.1–2007.12.31, Canada	3851 patients with localized PCa (T-stages included T1, T2, T3, and T4)		EBRT ± ADT	Post-primary treatment		8.4 years (median)	914	2937	(1) 60% (2) 70%	PCa-specific
Keskivali et al. 2016 [29]	Retrospective cohort, 1995–2009, Finland	1314 patients with PCa (pathologic stage including T1–T2 with N0 and T3 with or without N1)		RP	Pre- and post-primary treatment (19.1% pre-primary treatment)		8.6 years (median)	528	786	NR	All-cause; PCa-specific
Joentausta et al. 2019^†^ [30]	Retrospective cohort, 1995–2013, Finland	14,424 patients with localized PCa (N0 for localized, T3–T4 and all N1 for locally advanced)		RP	Pre- and post-diagnostic		(1) Pre-diagnostic: 6.33 years (median) (2) Post-diagnostic: 9.5 years (median)	(1) Pre-diagnostic: 3435 (2) Post-diagnostic: 6728	(1) Pre-diagnostic: 10,441 (2) Post-diagnostic: 7651	(1.1) 14.1% (1.2) 19.7% (2.1) 19.8% (2.2) 19.6%	PCa-specific ^₸^
Li et al. 2019 [31]	Retrospective cohort, 2000–2010, Taiwan	567 patients with PCa		RT	Pre- and/or Post-diagnostic		NR	174	213	NR	All-cause
Prabhu et al. 2021 [32]	Retrospective cohort, 2002–2015, USA	3088 patients with PCa (pathologic stage including T0, T2b–T2c, T3a–T4)		RP	Pre- and post-primary treatment		112.8 months (median)	1222	1866	NR	All-cause
**(B) Author, Year**	**All-Cause Mortality**			**Cancer-Specific Mortality**			**Adjusted For**				
	**HR**	**95% CI**	***p***	**HR**	**95% CI**	***p***					
Katz et al. 2010 * [8]	(1) 0.35 (2) 0.59	(1) 0.21–0.58 (2) 0.37–0.94	NR	NR	NR	NR	(1) Use of NSAIDs, mean office visits/year, age, cardiovascular disease, year of diagnosis, pathological T-stage (2) Use of NSAIDs, mean office visits/year, diabetes, Gleason score, current smoker at diagnosis				
Caon et al. 2014 [9]	NR	NR	NR	0.77	0.55–1.08	NR	Aspirin use, age, year of treatment, radiation dose, ADT use or not, PSA level, clinical T stage, CCI score, Gleason score				
Keskivali et al. 2016 ^‡^ [29]	(1) 1.08 (2) 1.28	(1) 0.69–1.69 (2) 0.92–1.78	NR	(1) 0.99 (2) 0.81	(1) 0.38–2.57 (2) 0.37–1.78	NR	Age, cancer stage, Gleason score, PSA level, surgical margin positivity, total cholesterol, and use of antidiabetic and antihypertensive drugs				
Joentausta et al. 2019 ^₸^ [30]	NR	NR	NR	(1) 0.70 (2) 0.83	(1) 0.52–0.95 (2) 0.67–1.03	NR	Age, cancer stage, any use of chemotherapy or radiotherapy for PCa, diabetes, hypertension, coronary artery disease, and obesity				
Li et al. 2019 ^§^ [31]	(1) 0.77 (2) 1.60 (3) 0.24	(1) 0.50–1.19 (2) 0.91–2.82 (3) 0.09–0.66	NR	NR	NR	NR	Age, diabetes, hypertension, cardiovascular disease, peripheral artery disease, and atherosclerosis				
Prabhu et al. 2021 [32]	1.30	0.97–1.74	0.077	NR	NR	NR	NR				

Abbreviations: ADT, androgen deprivation therapy; CCI, Charlson comorbidity index; CI, confidence interval; EBRT, external beam radiotherapy; NR, not reported; NSAIDs, non-steroidal anti-inflammatory drugs; PSA, prostate-specific antigen; RP, radical prostatectomy; RT, radiotherapy. * The median follow-up times for (1) RP and (2) RT patients were 44 months and 42 months, respectively. RP and RT patients were separately analyzed using multivariate Cox proportional hazards regression models. † (1) statin users; (2) non-statin users; (1.1) pre-diagnostic statin users; (1.2) pre-diagnostic non-statin users; (2.1) post-diagnostic statin users; (2.2) post-diagnostic non-statin users. ‡ Associations between statin use and mortality outcomes were reported for different times of statin use, including (1) statin use before a prostatectomy and (2) statin use after a prostatectomy. ₸ Associations between statin use and mortality outcomes were reported for different times of statin use, including (1) statin use before the PCa diagnosis and (2) statin use after the PCa diagnosis. § Associations between statin use and mortality outcomes were reported in patients (1) who had ever used statins and had different times of statin use, including (2) statin use before the PCa diagnosis and (3) statin use after the PCa diagnosis.

### 3.3. Quality Assessment of Included Studies

As no RCT reported the impact of statins combined with ADT or definitive therapy, we utilized the NOS to determine the quality of the included studies as shown in Supplementary Table S2. Eight factors were used to assess study quality according to the NOS. All observational studies included in our analysis were determined to be of high quality.

### 3.4. Statin Use and All-Cause Mortality

**Figure 2:**
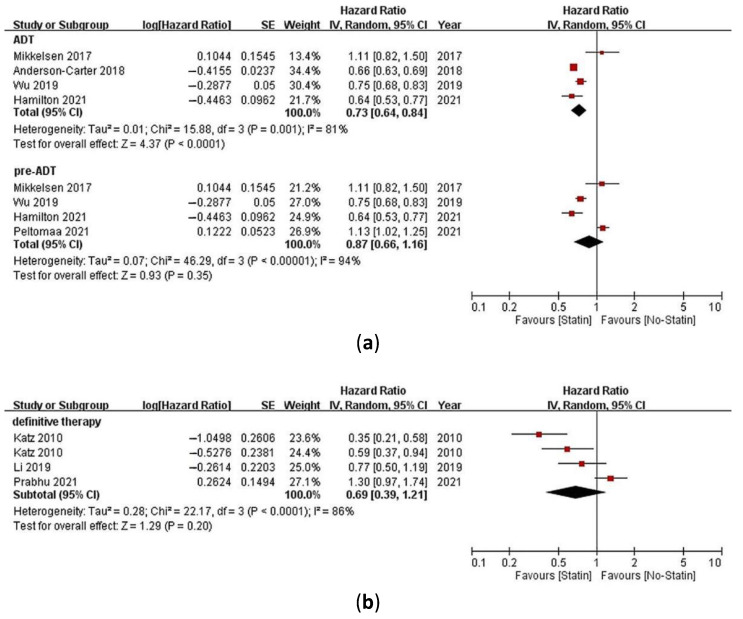
Meta-analysis of studies investigating (**a**) the association between statin use or statin use before androgen deprivation therapy (ADT) initiation (pre-ADT) with all-cause mortality of prostate cancer (PCa) patients receiving ADT as their primary treatment and (**b**) the association between statin use with all-cause mortality of PCa patients receiving definitive therapy as their primary treatment.

Five studies compared all-cause mortality in PCa patients receiving ADT as their primary treatment [23,24,25,26,27]. One of the studies (by Peltomaa et al.) separately reported statin use before and after ADT initiation [28]. Therefore, HRs with 95% CIs were pooled for the four other studies. The combined results showed that statin use significantly reduced the risk of all-cause mortality (HR = 0.73, 95% CI = 0.64–0.84, *p* < 0.0001, *I*^2^ = 81%, Figure 2a).

Among the five studies comparing all-cause mortality in PCa patients receiving ADT as their primary treatment [24,25,26,27,28], four of them reported the HRs of all-cause mortality for prevalent statin users (pre-ADT) [24,26,27,28]. The combined results showed that the pre-ADT use of statins was not significantly associated with a reduction in the risk of all-cause mortality (HR = 0.87, 95% CI = 0.66–1.16, *p* = 0.35, *I*^2^ = 94%, Figure 2a).

Four studies compared all-cause mortality in PCa patients receiving definitive therapy as their primary treatment [8,29,31,32]. One of the studies (by Keskivali et al.) separately reported statin use before and after a prostatectomy [29]. Furthermore, in one of the other three studies (by Katz et al.), the effects of statin use on all-cause mortality in PCa patients were provided separately in patients who underwent a radical prostatectomy or radiotherapy [8]. Therefore, four HRs with 95% CIs were pooled for these three studies. The combined results showed that statin use was not significantly associated with a reduction in the risk of all-cause mortality (HR = 0.69, 95% CI = 0.39–1.21, *p* = 0.20, *I*^2^ = 86%, Figure 2b).

### 3.5. Statin Use and Cancer-Specific Mortality

**Figure 3:**
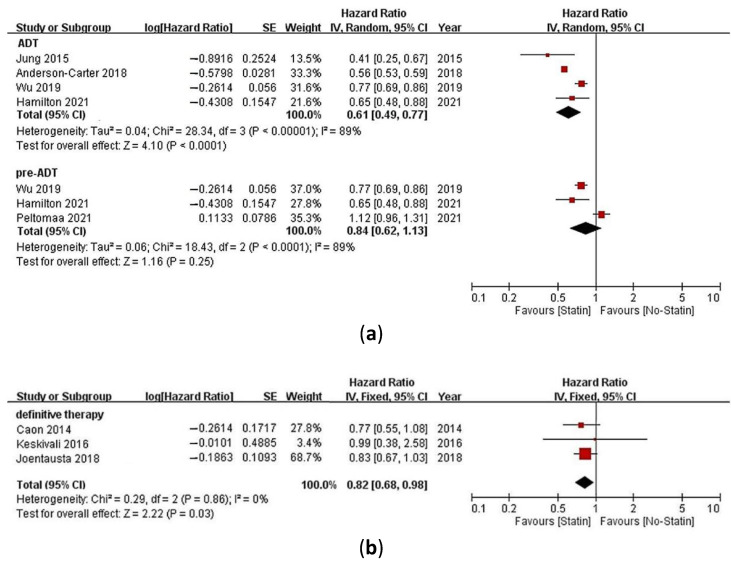
Meta-analysis of studies investigating (**a**) the association between statin use or statin use before androgen deprivation therapy (ADT) initiation (pre-ADT) with prostate cancer (PCa)-specific mortality of PCa patients receiving ADT as their primary treatment and (**b**) the association between statin use with PCa-specific mortality of PCa patients receiving definitive therapy as their primary treatment.

Five studies compared PCa-specific mortality in PCa patients receiving ADT as their primary treatment [23,25,26,27,28]. One of the studies (by Peltomaa et al.) separately reported statin use before and after ADT initiation [28]. Therefore, HRs with 95% CIs were pooled for the four other studies. The combined results showed that statin use significantly reduced the risk of PCa-specific mortality (HR = 0.61, 95% CI = 0.49–0.77, *p* < 0.0001, *I*^2^ = 89%, Figure 3a).

In the five studies comparing PCa-specific mortality in PCa patients receiving ADT as their primary treatment [23,25,26,27,28], three of them reported the HRs of PCa-specific mortality for prevalent statin users (pre-ADT) [26,27,28]. The combined results showed that the pre-ADT use of statins was not significantly associated with a reduction in the risk of PCa-specific mortality (HR = 0.84, 95% CI = 0.62–1.13, *p* = 0.25, *I*^2^ = 89%, Figure 3a).

Three studies compared PCa-specific mortality in PCa patients receiving definitive therapy as their primary treatment [9,29,30]. One of the studies (by Keskivali et al.) separately reported statin use before and after a prostatectomy [29], whereas another of the studies (by Joentausta et al.) separately reported statin use before and after the PCa diagnosis [30]. Accordingly, the highest HRs with 95% CIs of the three studies were pooled together in the main analysis. The combined results showed that statin use significantly reduced the risk of PCa-specific mortality (HR = 0.82, 95% CI = 0.68–0.98, *p* = 0.03, *I*^2^ = 0%, Figure 3b).

### 3.6. Sensitivity Analysis

To evaluate the effect of statin use on all-cause mortality and PCa-specific mortality, sensitivity analyses were conducted in PCa patients receiving definitive therapy as their primary treatment. For all-cause mortality, the five highest HRs and five lowest HRs from four studies were pooled [8,29,31,32]. The combined results showed that statin was not significantly associated with a reduction in the risk of all-cause mortality in either the sensitivity analysis with the highest HRs (HR 0.90, 95% CI 0.55–1.48, *p* = 0.03, *I*^2^ = 86%, Supplementary Figure S1a) or that with the lowest HRs (HR = 0.90, 95% CI = 0.55–1.48, *p* = 0.03, *I*^2^ = 86%, Supplementary Figure S1a).

For PCa-specific mortality, the three lowest HRs from three studies were pooled in the sensitivity analysis [9,29,30]. The combined results showed that statin use significantly reduced the risk of PCa-specific mortality (HR = 0.74, 95% CI = 0.59–0.91, *p* = 0.005, *I*^2^ = 0%, Supplementary Figure S1b).

### 3.7. Discussion

This meta-analysis addressed the relationship between mortality and statin use in PCa patients receiving ADT or definitive therapy as their primary treatment at the same time. Our results showed that statin use was associated with a reduced risk of PCa-specific mortality in patients receiving ADT or definitive treatment. However, the survival benefit of all causes of death from statin use was only observed in patients receiving ADT. Moreover, we studied the effect of the timing of statin initiation on mortality and did not find a survival benefit in patients who had initiated statin use before receiving ADT.

Our meta-analysis did not show that prevalent statin users, PCa patients who had initiated statins before receiving ADT, had better all-cause mortality or PCa-specific mortality compared to non-statin users. This discrepancy in mortality between prevalent and new statin users might be explained by selection bias. Compared to non-statin users, prevalent users may have had different characteristics that affected their survival. Despite these observational studies adjusting for risk factors associated with mortality, residual confounding may still occur and cause differences in the effects of statins on mortality among studies. However, statin use was associated with a reduced mortality risk in advanced PCa patients receiving ADT. Collectively, our results indicated that the benefit of statin use in advanced PCa patients receiving ADT might come from initiating statin use after ADT. Corresponding to our results, an ongoing phase 3 clinical trial (NCT04026230) aimed to evaluate the effect of statin use after ADT initiation on mortality in advanced PCa patients receiving ADT. The trial results may potentially resolve the question of whether statins have a positive effect on PCa outcomes.

A prior clinical trial showed that atorvastatin did not lower the PCa proliferation rate compared to a placebo in PCa patients treated with a radical prostatectomy [33]. Notably, that clinical trial only focused on biochemical outcomes, such as intraprostatic Ki-67 and serum prostate-specific antigen (PSA) changes, with short-term statin use and follow-up. Three of our included observational studies reported the effect of statin use on PCa-specific mortality in patients receiving definitive therapy, with at least 6 years of median follow-up. Although all three studies showed a neutral effect, the combined estimate in our meta-analysis suggested a significant beneficial effect of statin use on PCa-specific mortality with no evidence of heterogeneity. Only a few studies investigated the association between statin use and cancer-specific mortality in PCa patients receiving definitive therapy. Thus, our meta-analysis conservatively pooled the highest HRs for each study as the main analysis. The results of the sensitivity analysis that pooled the lowest HRs in each study demonstrated a more substantial benefit. In contrast to cancer-specific mortality, our meta-analysis did not show a significant beneficial effect of statin use on all-cause mortality in PCa patients receiving definitive therapy. Given that the 10-year survival in men with early-stage PCa is greater than 90% [34], the benefit gained from statin use in patients with early-stage PCa receiving definitive therapy was too small to demonstrate statistical significance.

Several possible mechanisms may account for the positive effect of statins on PCa, which include inhibition of the proteasome pathway [35], reduction in cholesterol synthesis [36], initiation of tumor-specific apoptosis [37], and anti-inflammatory and antiangiogenic effects [38,39]. As cholesterol is the precursor of androgen production, statins contributing to reducing testosterone and dihydrotestosterone synthesis could lead to a reduction in androgen-dependent PCa tumor growth [2,40]. In addition to the reduction in cholesterol formation, statins reduce androgen sensitivity and cell proliferation by decreasing the protein expression levels of androgen receptors in PCa cells [41]. In line with this, our study suggested that statin use was associated with better mortality in both PCa patients receiving ADT or definitive therapy. However, all-cause mortality did not show significance in PCa patients receiving definitive therapy.

The findings of our meta-analysis should be carefully interpreted due to the following limitations: First, there could be a potential immortal time bias, as some of the cohort studies included a time-fixed exposure to define statin use; that is, exposure to statins was defined irrespective of when statin use truly began. By this definition, statin-unexposed follow-up time would be misclassified as exposed time. Thus, PCa patients in the statin-exposed group may inherently have been given a survival advantage. This misclassification would have falsely exaggerated the effect of statin use on mortality among PCa patients. Second, as we know, statins can be categorized into two types, namely, hydrophilic statins (fluvastatin, pravastatin, and rosuvastatin) and lipophilic statins (atorvastatin, simvastatin, pitavastatin, and cerivastatin). Moreover, the effect of statins on mortality in PCa patients may depend on the type [42,43,44]. However, due to our meta-analysis, which pooled both types of statins together, this study could not conclude which type of statin or specific drug would have a stronger beneficial effect. Third, this meta-analysis could not provide evidence for the impact of pre-definitive therapy or post-definitive therapy statin use on mortality in PCa patients because the timing of statin initiation varied across the six included studies. Within the six studies, some patients began using statins before or after definitive therapy, while others began using statins before or after a PCa diagnosis. Finally, the differences in time, dose, and duration of statins among the included studies could be a potential cause for the heterogeneity in the pooled study estimates.

The findings and limitations of our study suggested the scope for future clinical trials to evaluate the effects of statins combined with primary therapy in PCa patients. Statins are relatively safer and cheaper than chemotherapy and newer generation anti-androgens. Therefore, the beneficial effects associated with statins make them good candidates for additional therapy to standard treatment to manage PCa.

## 4. Conclusions

Our meta-analysis suggested that statin use in combination with ADT was associated with better all-cause and cancer-specific mortality in PCa patients; however, the beneficial effect might not come from statin use before ADT initiation. Furthermore, we demonstrated that statin use in combination with definitive therapy did not lower the risk of all-cause mortality in PCa patients, although improved cancer-specific mortality was found in this population.

## Data Availability

No new data were created or analyzed in this study. Data sharing is not applicable to this article.

## Supplementary Materials

The following supporting information can be downloaded at: https://www.mdpi.com/article/10.3390/ph15020131/s1, Table S1: Newcastle–Ottawa Scale quality assessment of the included observational studies, Table S2: Syntax used in the database searches; Figure S1: Sensitivity analysis of studies investigating the association between statin use with (a) all-cause mortality and (b) prostate cancer (PCa)-specific mortality of PCa patients receiving definitive therapy as their primary treatment.

## References

[1] Hu M., Cheung B.M.Y., Tomlinson B. (2012). Safety of statins: An update. Ther. Adv. Drug Saf..

[2] Papadopoulos G., Delakas D., Nakopoulou L., Kassimatis T. (2011). Statins and prostate cancer: Molecular and clinical aspects. Eur. J. Cancer (Oxf. Engl. 1990).

[3] Hamilton R.J., Freedland S.J. (2008). Rationale for statins in the chemoprevention of prostate cancer. Curr. Urol. Rep..

[4] Lifshitz K., Ber Y., Margel D. (2021). Role of Metabolic Syndrome in Prostate Cancer Development. Eur. Urol. Focus.

[5] Shoag J., Barbieri C.E. (2016). Clinical variability and molecular heterogeneity in prostate cancer. Asian J. Androl..

[6] Marcella S.W., David A., Ohman-Strickland P.A., Carson J., Rhoads G.G. (2012). Statin use and fatal prostate cancer: A matched case-control study. Cancer.

[7] Niraula S., Pond G., de Wit R., Eisenberger M., Tannock I.F., Joshua A.M. (2013). Influence of concurrent medications on outcomes of men with prostate cancer included in the TAX 327 study. Can. Urol. Assoc. J..

[8] Katz M.S., Carroll P.R., Cowan J.E., Chan J.M., D’Amico A.V. (2010). Association of statin and nonsteroidal anti-inflammatory drug use with prostate cancer outcomes: Results from CaPSURE. BJU Int..

[9] Caon J., Paquette M., Hamm J., Pickles T. (2014). Does statin or ASA affect survival when prostate cancer is treated with external beam radiation therapy?. Prostate Cancer.

[10] Emilsson L., García-Albéniz X., Logan R.W., Caniglia E.C., Kalager M., Hernán M.A. (2018). Examining bias in studies of statin treatment and survival in patients with cancer. JAMA Oncol..

[11] Sung H., Ferlay J., Siegel R.L., Laversanne M., Soerjomataram I., Jemal A., Bray F. (2021). Global Cancer Statistics 2020: GLOBOCAN Estimates of Incidence and Mortality Worldwide for 36 Cancers in 185 Countries. CA A Cancer J. Clin..

[12] Keyes M., Crook J., Morton G., Vigneault E., Usmani N., Morris W.J. (2013). Treatment options for localized prostate cancer. Can. Fam. Physician.

[13] Saad F., Fizazi K. (2015). Androgen Deprivation Therapy and Secondary Hormone Therapy in the Management of Hormone-sensitive and Castration-resistant Prostate Cancer. Urology.

[14] Meng Y., Liao Y.-B., Xu P., Wei W.-R., Wang J. (2016). Statin use and mortality of patients with prostate cancer: A meta-analysis. OncoTargets Ther..

[15] Park H.S., Schoenfeld J.D., Mailhot R.B., Shive M., Hartman R.I., Ogembo R., Mucci L.A. (2013). Statins and prostate cancer recurrence following radical prostatectomy or radiotherapy: A systematic review and meta-analysis. Ann. Oncol. Off. J. Eur. Soc. Med. Oncol..

[16] Yang H., Pang L., Hu X., Wang W., Xu B., Zhang X., Liu L. (2020). The effect of statins on advanced prostate cancer patients with androgen deprivation therapy or abiraterone/enzalutamide: A systematic review and meta-analysis. J. Clin. Pharm. Ther..

[17] Moher D., Liberati A., Tetzlaff J., Altman D.G., Group P. (2009). Preferred Reporting Items for Systematic Reviews and Meta-Analyses: The PRISMA Statement. PLoS Med..

[18] Wells G.A., Shea B., O’Connell D., Peterson J., Welch V., Losos M., Tugwell P. (2000). The Newcastle-Ottawa Scale (NOS) for Assessing the Quality of Nonrandomised Studies in Meta-Analyses. http://www.ohri.ca/programs/clinical_epidemiology/oxford.asp.

[19] Lo C.K.-L., Mertz D., Loeb M. (2014). Newcastle-Ottawa Scale: Comparing reviewers’ to authors’ assessments. BMC Med. Res. Methodol..

[20] Van Houwelingen H.C., Arends L.R., Stijnen T. (2002). Advanced methods in meta-analysis: Multivariate approach and meta-regression. Stat. Med..

[21] Higgins J.P., Thompson S.G., Deeks J.J., Altman D.G. (2003). Measuring inconsistency in meta-analyses. BMJ.

[22] Peters J.L., Sutton A.J., Jones D.R., Abrams K.R., Rushton L. (2008). Contour-enhanced meta-analysis funnel plots help distinguish publication bias from other causes of asymmetry. J. Clin. Epidemiol..

[23] Jung J., Lee C., Lee C., Kwon T., You D., Jeong I.G., Hong J.H., Ahn H., Kim C.-S. (2015). Effects of statin use on the response duration to androgen deprivation therapy in metastatic prostate cancer. Korean J. Urol..

[24] Mikkelsen M.K., Thomsen F.B., Berg K.D., Jarden M., Larsen S.B., Hansen R.B., Brasso K. (2017). Associations between statin use and progression in men with prostate cancer treated with primary androgen deprivation therapy. Scand. J. Urol..

[25] Anderson-Carter I., Posielski N., Liou J.I., Khemees T.A., Downs T.M., Abel E.J., Jarrard D.F., Richards K.A. (2019). The impact of statins in combination with androgen deprivation therapyin patients with advanced prostate cancer: A large observational study. Urol. Oncol. Semin. Orig. Investig..

[26] Wu S.-Y., Fang S.-C., Shih H.-J., Wen Y.-C., Shao Y.-H.J. (2019). Mortality associated with statins in men with advanced prostate cancer treated with androgen deprivation therapy. Eur. J. Cancer.

[27] Hamilton R.J., Ding K., Crook J.M., O’Callaghan C.J., Higano C.S., Dearnaley D.P., Horwitz E.M., Goldenberg S.L., Gospodarowicz M.K., Klotz L. (2021). The association between statin use and outcomes in patients initiating androgen deprivation therapy. Eur. Urol..

[28] Peltomaa A., Raittinen P., Talala K., Taari K., Tammela T., Auvinen A., Murtola T. (2021). Prostate cancer prognosis after initiation of androgen deprivation therapy among statin users. A population-based cohort study. Prostate Cancer Prostatic Dis..

[29] Keskiväli T., Kujala P., Visakorpi T., Tammela T.L., Murtola T.J. (2016). Statin use and risk of disease recurrence and death after radical prostatectomy. Prostate.

[30] Joentausta R.M., Rannikko A., Murtola T.J. (2019). Prostate cancer survival among statin users after prostatectomy in a Finnish nationwide cohort. Prostate.

[31] Li K., Si-Tu J., Qiu J., Lu L., Mao Y., Zeng H., Chen M., Lai C., Chang H.-J., Wang D. (2019). Statin and metformin therapy in prostate cancer patients with hyperlipidemia who underwent radiotherapy: A population-based cohort study. Cancer Manag. Res..

[32] Prabhu N., Kapur N., Catalona W., Leikin R., Helenowski I., Jovanovich B., Gurley M., Okwuosa T.M., Kuzel T.M. (2021). Statin use and risk of prostate cancer biochemical recurrence after radical prostatectomy. Urol. Oncol. Semin. Orig. Investig..

[33] Murtola T.J., Syvälä H., Tolonen T., Helminen M., Riikonen J., Koskimäki J., Pakarainen T., Kaipia A., Isotalo T., Kujala P. (2018). Atorvastatin versus placebo for prostate cancer before radical prostatectomy—A randomized, double-blind, placebo-controlled clinical trial. Eur. Urol..

[34] Hamdy F.C., Donovan J.L., Lane J., Mason M., Metcalfe C., Holding P., Davis M., Peters T.J., Turner E.L., Martin R.M. (2016). 10-year outcomes after monitoring, surgery, or radiotherapy for localized prostate cancer. N. Engl. J. Med..

[35] Rao S., Porter D.C., Chen X., Herliczek T., Lowe M., Keyomarsi K. (1999). Lovastatin-mediated G1 arrest is through inhibition of the proteasome, independent of hydroxymethyl glutaryl-CoA reductase. Proc. Natl. Acad. Sci. USA.

[36] Jones P.H., Davidson M.H., Stein E.A., Bays H.E., McKenney J.M., Miller E., Cain V.A., Blasetto J.W., Group S.S. (2003). Comparison of the efficacy and safety of rosuvastatin versus atorvastatin, simvastatin, and pravastatin across doses (STELLAR Trial). Am. J. Cardiol..

[37] Wong W.W., Dimitroulakos J., Minden M., Penn L. (2002). HMG-CoA reductase inhibitors and the malignant cell: The statin family of drugs as triggers of tumor-specific apoptosis. Leukemia.

[38] Dulak J., Józkowicz A. (2005). Anti-angiogenic and anti-inflammatory effects of statins: Relevance to anti-cancer therapy. Curr Cancer Drug Targets.

[39] Pelton K., Freeman M.R., Solomon K.R. (2012). Cholesterol and prostate cancer. Curr. Opin. Pharmacol..

[40] Roy M., Kung H.-J., Ghosh P.M. (2011). Statins and prostate cancer: Role of cholesterol inhibition vs. prevention of small GTP-binding proteins. Am. J. Cancer Res..

[41] Yokomizo A., Shiota M., Kashiwagi E., Kuroiwa K., Tatsugami K., Inokuchi J., Takeuchi A., Naito S. (2011). Statins reduce the androgen sensitivity and cell proliferation by decreasing the androgen receptor protein in prostate cancer cells. Prostate.

[42] Boudreau D.M., Yu O., Buist D.S., Miglioretti D.L. (2008). Statin use and prostate cancer risk in a large population-based setting. Cancer Causes Control.

[43] Agalliu I., Salinas C.A., Hansten P.D., Ostrander E.A., Stanford J.L. (2008). Statin use and risk of prostate cancer: Results from a population-based epidemiologic study. Am. J. Epidemiol..

[44] Shannon J., Tewoderos S., Garzotto M., Beer T.M., Derenick R., Palma A., Farris P.E. (2005). Statins and prostate cancer risk: A case-control study. Am. J. Epidemiol..

