# Supplementary material for: The Effects of Statins on Prostate Cancer Patients Receiving Androgen Deprivation Therapy or Definitive Therapy: A Systematic Review and Meta-Analysis
Source: Pharmaceuticals (Basel). 2022 Jan 22;15(2):131. doi: 10.3390/ph15020131 (PMC8875347; doi:10.3390/ph15020131)
Supplement: Supplementary file 1 [file pharmaceuticals-15-00131-s001.zip › pharmaceuticals-1551062-supplementary.pdf]

Table S1. Syntax used in the database searches

Database	Search strategy	Results
Cochrane Library	(prostate cancer) OR (statin)	76 references
Embase	('prostate cancer'/exp OR 'prostate cancer' OR 'malignant prostate tumor' OR 'malignant prostate tumour' OR 'malignant prostatic tumor' OR 'malignant prostatic tumour' OR 'prostate gland cancer' OR 'prostate malignancy' OR 'prostate malignant neoplasm' OR 'prostate malignant tumor' OR 'prostate malignant tumour' OR 'prostatic cancer' OR 'prostatic malignancy') AND ('hydroxymethylglutaryl coenzyme a reductase inhibitor'/exp OR 'hydroxymethylglutaryl coenzyme a reductase inhibitor' OR 'hmg coa reductase inhibitor' OR 'hmg coa reductase inhibitors' OR 'hmg coenzyme a reductase inhibitor' OR 'hmg-coa reductase inhibitors' OR 'hydroxymethylglutaryl coa reductase inhibitors' OR 'hydroxymethylglutaryl-coa reductase inhibitors' OR 'statin' OR 'statins' OR 'vastatin' OR 'simvastatin' OR 'lovastatin' OR 'pravastatin' OR 'fluvastatin' OR 'atorvastatin' OR 'cerivastatin' OR 'rosuvastatin' OR 'pitavastatin') AND ('radiotherapy'/exp OR 'radiotherapy' OR ' bioradiant therapy' OR ' bucky irradiation' OR ' bucky radiation' OR ' bucky radiotherapy' OR ' bucky ray' OR ' bucky ray radiation' OR ' bucky therapy' OR ' fractionated radiotherapy' OR ' hemibody irradiation' OR ' hypophysis irradiation' OR ' hypophysis radiation ' OR ' irradiation therapy ' OR ' irradiation treatment ' OR ' lymphatic irradiation ' OR ' pituitary irradiation ' OR ' radiation beam centration ' OR ' radiation repair ' OR ' radiation therapy ' OR ' radiation treatment ' OR ' radio therapy ' OR ' radio treatment ' OR ' radiohypophysectomy ' OR ' radiotherapy setup errors ' OR ' radiotreatment ' OR ' roentgen therapy ' OR ' roentgen treatment ' OR ' rontgen therapy ' OR ' therapeutic radiology ' OR ' x radiotherapy ' OR ' x ray therapy ' OR ' x ray treatment ' OR ' x-ray therapy ' OR 'External beam radiation therapy' or 'External beam radiation therapies' or 'External beam radiation treatment' or 'External beam radiation treatments':ab,ti or 'external beam radiation' or 'external beam radiotherapy' or 'external beam radiotherapies' or 'external beam therapy' or 'external beam therapies' or 'external beam treatment' or 'external beam treatments' or 'EBRT' OR 'Internal radiation therapy' or 'Internal radiation therapies' or 'Internal radiation treatment' or 'Internal radiation treatments' or 'brachytherapy' or 'brachytherapies' or 'internal radiotherapy' or 'internal radiotherapies' or 'IRT' OR 'prostatectomy'/exp OR 'prostatectomy' OR 'prostate adenectomy ' OR 'prostate resection ' OR 'prostatic adenectomy ' OR 'radical prostatectomy ' OR 'total prostatectomy ' OR 'androgen deprivation therapy'/exp OR 'androgen deprivation	736 references

	therapy' OR 'adt' OR 'androgen suppression therapy' OR 'hormone therapy' OR 'androgen deprivation treatment' OR 'ast' OR 'androgen suppression treatment')	
PubMed	("Prostatic Neoplasms"[Mesh] OR "Prostate Neoplasms" or "Prostate Neoplasm" or "Prostatic Neoplasm" or "Prostate Cancer" or "Prostate Cancers" or "Cancer of the Prostate" or "Prostatic Cancer" or "Prostatic Cancers" or "Cancer of Prostate") AND ("Hydroxymethylglutaryl-CoA Reductase Inhibitors"[Mesh] OR "Hydroxymethylglutaryl CoA Reductase Inhibitors" or "HMG-CoA Reductase Inhibitor" or "HMG CoA Reductase Inhibitor" or "Statin" or "HMG-CoA Statins" or "HMG-CoA Reductase Inhibitors" or "HMG CoA Reductase Inhibitors" or "Hydroxymethylglutaryl-Coenzyme A Inhibitors" or "Hydroxymethylglutaryl-CoA Inhibitors" or "Hydroxymethylglutaryl-CoA Reductase Inhibitor" or "Hydroxymethylglutaryl CoA Reductase Inhibitor" or "Statins" or "simvastatin" or "lovastatin" or "pravastatin" or "fluvastatin" or "atorvastatin" or "cerivastatin" or "rosuvastatin"or "pitavastatin") AND ("Radiotherapy"[Mesh] OR "Radiotherapy" or "Radiotherapies" or "Radiation Therapy" or "Radiation Therapies" or "Radiation Treatment" or "Radiation Treatments" or "Targeted Radiotherapies" or "Targeted Radiotherapy" or "Targeted Radiation Therapy" or "Targeted Radiation Therapies" OR "External beam radiation therapy" or "External beam radiation therapies" or "External beam radiation treatment" or "External beam radiation treatments" or "external beam radiation" or "external beam radiotherapy" or "external beam radiotherapies" or "external beam therapy" or "external beam therapies" or "external beam treatment" or "external beam treatments" or "EBRT" OR "Internal radiation therapy" or "Internal radiation therapies" or "Internal radiation treatment" or "Internal radiation treatments" or "brachytherapy" or "brachytherapies" or "internal radiotherapy" or "internal radiotherapies" or "IRT" OR "Prostatectomy"[Mesh] OR "Prostatectomy" or "Prostatectomies" or "Suprapubic Prostatectomies" or "Suprapubic Prostatectomy" or "Retropubic Prostatectomies" or "Retropubic Prostatectomy" or "Definitive therapy" or " Definitive therapies" OR "Androgen deprivation therapy" or "androgen suppression therapy" or "ADT" or "AST" or "Androgen deprivation treatment" or "androgen suppression treatment" or "Hormone therapy")	164 references

Table S2. Newcastle-Ottawa Scale quality assessment of the included observational studies

	Selection				Comparability	Outcome			
Author, year	①	②	③	④	⑤	⑥	⑦	⑧	Total
Katz et al., 2010	★	★		★	★★	★	★	★	8
Caon et al., 2014	★	★	★	★	★★	★	★	★	9
Jung et al., 2015		★	★	★	★★	★	★	★	8
Keskivali et al., 2016	★	★	★	★	★★	★	★	★	9
Mikkelsen et al., 2017	★	★	★	★	★★	★	★	★	9
Anderson-Carter et al., 2018	★	★	★	★	★★	★	★	★	9
Joentausta et al., 2019	★	★	★	★	★★	★	★	★	9
Li et al., 2019	★	★	★	★	★★	★	★		8
Wu et al., 2019	★	★	★	★	★★	★	★	★	9
Prabhu et al., 2020	★	★	★	★		★	★	★	7
Hamilton et al., 2021		★		★	★★	★	★	★	7
Peltomaa et al., 2021	★	★	★	★	★★	★	★	★	9

Notes. ①: representativeness of the exposed cohort; ②: selection of the non-exposed cohort; ③: ascertainment of exposure; ④: demonstration that the outcome of interest was not present at the start of the study; ⑤: comparability of cohorts on the basis of the design or analysis; ⑥: assessment of the outcome; ⑦: was follow-up long enough for outcomes to occur?; ⑧: adequacy of follow-up of cohorts

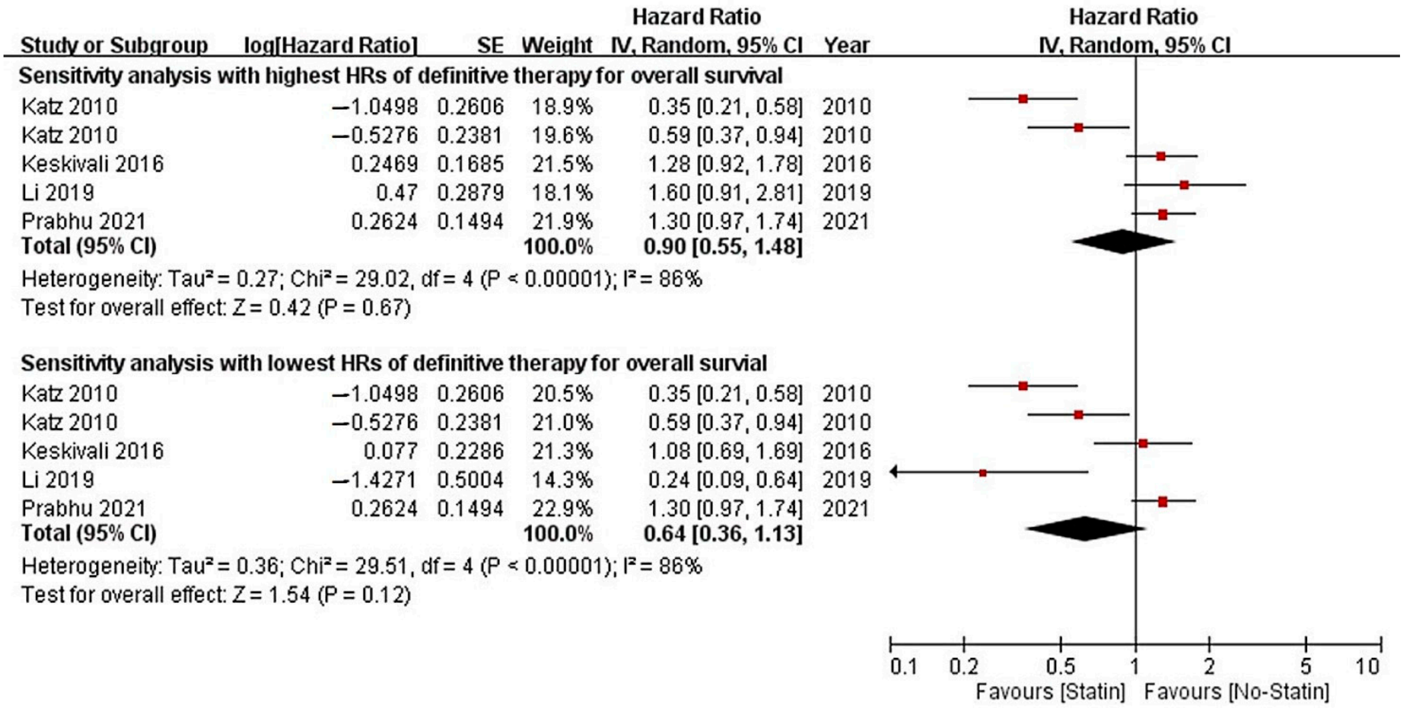

(**a**)

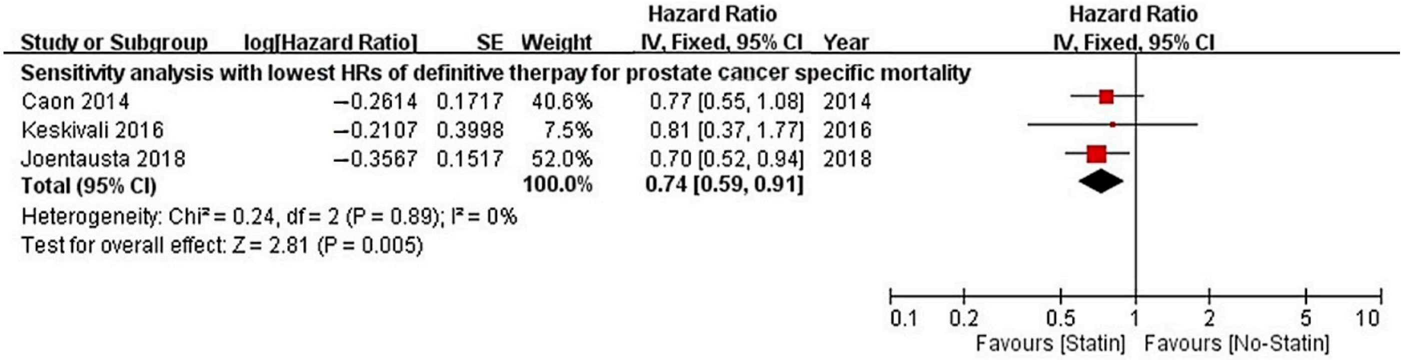

(**b**)

Figure S1. Sensitivity analysis of studies investigating the association between statin use with (a) **all-cause mortality** and (b) **prostate cancer (PCa)-specific mortality** of PCa patients receiving definitive therapy as their primary treatment.
